# Effectiveness of a Manual Therapy Protocol in Women with Pelvic Pain Due to Endometriosis: A Randomized Clinical Trial

**DOI:** 10.3390/jcm12093310

**Published:** 2023-05-06

**Authors:** Elena Muñoz-Gómez, Ana María Alcaraz-Martínez, Sara Mollà-Casanova, Núria Sempere-Rubio, Marta Aguilar-Rodríguez, Pilar Serra-Añó, Marta Inglés

**Affiliations:** 1 Research Unit in Clinical Biomechanics (UBIC), Department of Physiotherapy, Faculty of Physiotherapy, University of Valencia, 46010 Valencia, Spain; elena.munoz-gomez@uv.es (E.M.-G.); sara.molla@uv.es (S.M.-C.); marta.aguilar@uv.es (M.A.-R.); pilar.serra@uv.es (P.S.-A.); marta.ingles@uv.es (M.I.); 2 Fisio Espai Albal, Physical Therapy Center, 46470 Valencia, Spain; alcarazanam@gmail.com

**Keywords:** manual therapy, endometriosis, pelvic pain, health profile, quality of life, emotional condition

## Abstract

A randomized controlled trial was carried out to assess the effectiveness of a manual therapy protocol in terms of the clinical characteristics, quality of life, and emotional condition of the women with endometriosis-related pelvic pain. Forty-one women (mean age of 36.10 (6.97) years) with pelvic pain due to endometriosis were randomly divided into (i) a manual therapy group (MTG) (*n* = 21) and (ii) a placebo group (PG) (*n* = 20). Both groups received an 8-week intervention. Pain, lumbar mobility, endometriosis health profile, quality of life, depression and anxiety levels, and the patient’s perception of change were assessed before (T0) and after (T1) the intervention, as well as at a one-month follow-up (T2) and a six-month follow-up (T3). The MTG significantly improved pain intensity, powerlessness, lumbar mobility, and physical quality of life at T1 (*p* < 0.05). The results were maintained for pain intensity at T2 and T3. In addition, both the MTG and PG improved emotional wellbeing at T1 (*p* < 0.05). Neither group improved in terms of social support, self-image, and depression and anxiety levels after the intervention (*p* > 0.05). In conclusion, manual therapy may be an excellent complement to the gynecological treatment of endometriosis-related pelvic pain by alleviating pain and improving women’s endometriosis health profile and physical quality of life.

## 1. Introduction

Endometriosis is a debilitating condition with features of chronic inflammation that affects 10–15% of women of reproductive age [1]. One of the most common symptoms in women with endometriosis is chronic pelvic pain [2], defined as non-cyclic pain perceived in structures related to the pelvis and present for at least 6 months [3], which in turn affects the quality of life of those affected [4]. In addition, women with endometriosis, especially those with chronic pelvic pain, also have an increased vulnerability to psychological disorders such as depression and anxiety [5,6].

Treatment for endometriosis includes hormone treatment and surgery (laparoscopy or an eventual hysterectomy). However, pelvic pain associated with endometriosis is often refractory to them [7,8]. Since the pain often persists after medical treatment and the intensity of the pain does not correlate with tissue injury, the activation of peripheral nociceptors, or evidence of damage to neuronal somatosensory pathways or structures, it has been classified as nociplastic pain [6,9], suggesting that other mechanisms may continue to generate pain without the need for peripheral input [10]. In this regard, central sensibilization and myofascial dysfunction have been proposed to be involved in the initiation, amplification, and perpetuation of chronic pelvic pain [11]; thus, the search for other treatment options to relieve pain in this population is a challenge of paramount importance. Furthermore, as a chronic pain condition, women with endometriosis may have decreased physical activity and experience physical deconditioning, which, in turn, may cause musculoskeletal consequences. Indeed, a poorer physical status, such as lower lumbopelvic mobility and increased fatigue, have been found in women with endometriosis when compared with healthy women [12], which may negatively impact their daily living activities.

In this context, physiotherapy can contribute to the multidisciplinary assessment and treatment of pelvic pain due to endometriosis [13]. Physiotherapy includes passive therapies with no active participation by the patient (i.e., manual therapy, acupuncture or dry needling, heat, cold, electrical currents) and active therapies involving the patients’ joint and muscle movement as part of the therapy (i.e., therapeutic physical exercise) [14]. In this regard, manual therapy is a non-fatiguing intervention that could improve some aspects related to central sensibilization, such as inhibitory pain regulation and neuronal excitability in the dorsal horn of the medulla, in patients with chronic pain [15]. Furthermore, it is considered to be a well-tolerated treatment by patients [16]. Manual therapy techniques include soft tissue mobilization, joint mobilization/manipulation, nerve manipulation, and acupressure techniques [17]. In this context, we have reported that a manual therapy protocol consisting of articulatory techniques and soft tissue techniques was able to improve pain and clinical features related to quality of life in people with chronic pain due to migraine [18,19], with better results when applied together [20]. Some prospective pilot studies have applied manual therapy based on soft tissue and articulatory techniques in patients with pelvic pain due to endometriosis, and the studies have shown a trend towards an improvement in pain and quality of life [21,22,23]. However, these studies did not include any control group and the samples were too small (maximum of 20 patients). In addition, to date, no randomized controlled trial has investigated whether the application of a manual therapy protocol combining articulatory and soft tissue techniques improved lumbar mobility, the endometriosis health profile, or the emotional condition of people with endometriosis.

Thus, the aim of this study was to analyze the effectiveness of a manual therapy protocol in relation to the pelvic pain, lumbar mobility, and clinical features related to quality of life (i.e., endometriosis health profile and self-perception of quality of life) and the emotional condition (i.e., depression and anxiety levels) of women who suffer from pelvic pain due to endometriosis.

## 2. Materials and Methods

### 2.1. Participants

Participants diagnosed with endometriosis who suffered from chronic pelvic pain were recruited as a convenience sample from primary care centers in Valencia (Spain) (November to December 2021). All participants were recruited in the same way and all potential participants were provided with the same information about the study. The inclusion criteria were pre-menopausal woman between 18 and 50 years old with an endometriosis diagnosis from a gynecologist and associated pelvic pain for at least one year of evolution. The exclusion criteria were being pregnant, having rheumatic or degenerative neurological diseases, or having another injury or disease that caused pelvic pain, as well as having undergone any pelvic surgery within the previous year or receiving physical therapy treatment within the previous three months.

### 2.2. Study Design

A randomized controlled trial was carried out (NCT05418751) from January to December 2022 at a clinical center. The sample was randomly divided into two groups: (i) the manual therapy group (MTG), which received a manual therapy protocol, and (ii) the placebo group (PG), who received a hands-on placebo intervention. Evaluations were performed at four time points: (i) before the intervention (T0), (ii) after the intervention (T1) (two months after the start of treatment), (iii) at the one-month follow-up (T2), and (iv) at the six-month follow-up (T3).

All participants provided written informed consent, all procedures were conducted in accordance with the ethical standards of the Declaration of Helsinki, and all protocols were approved by the Ethics Committee of the University of Valencia (num. 2063942).

### 2.3. Randomization, Blinding, and Masking

The randomized allocation was performed through an online randomization tool (www.randomized.com accessed on 10 January 2022) by an external investigator who was not involved in the assessment or treatment of the participants. The participants were blinded to the treatment allocation. In addition, the outcome assessors were blinded to the study hypothesis, the intervention, and the treatment group assignment, thus avoiding researcher bias. The only researcher who was aware of treatments was the physiotherapist who performed manual therapy and placebo interventions.

### 2.4. Interventions

The intervention was carried out by a physiotherapist with more than 10 years of clinical experience in physiotherapy, of which 5 years were in pelvic floor physiotherapy. The interventions lasted for 8 weeks, with one session for 30 min every 15 days. In order to avoid the experimenter expectancy effect bias, the physiotherapist was blinded to the study hypothesis and outcome measures.

#### 2.4.1. Manual Therapy Protocol

**Figure 1:**
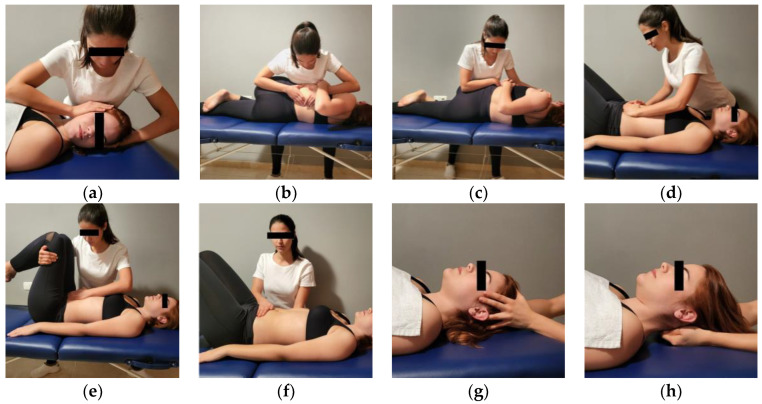
Manual therapy protocol. (**a**) Occiput, atlas, and axis manipulation technique. (**b**) Thoraco-lumbar manipulation technique. (**c**) Global sacroiliac manipulation technique. (**d**) Abdominal mobilization technique. (**e**) Broad ligament mobilization technique. (**f**) Pelvic diaphragm release technique. (**g**) Sphenoid technique. (**h**) Fourth ventricle technique.

The participants received a manual therapy protocol consisting of the combination of soft tissue and articulatory techniques based on previous studies [21,22,23] (Figure 1):

Occiput, atlas, and axis (OAA) manipulation technique: The patient was in the supine position. Manipulation was performed on a vertical axis (without flexion or extension, and very little lateral flexion) passing through the odontoid process of the axis. Prior to manipulation, slight cephalic decompression and small circumductions were performed. A rotation toward the side to be manipulated was performed with a cranial helical movement. This technique was applied bilaterally [24].

Thoraco-lumbar manipulation technique: The patient was laid on the contralateral side to be treated, with the upper and lower limbs flexed leading to a complete spine rotation, focusing on the T12-L1 junction. The therapist’s caudal hand pressed on the inferior articular apophyses of T12, while their cranial hand rested on the chest of the patient. A high-velocity low-amplitude movement in forward rotation of the pelvis was then performed. This technique was applied bilaterally [25].

Global sacroiliac manipulation technique: The patient was laid on the contralateral side to be treated. Adjustment was effected through complete spine rotation and lower limb flexion, focusing on the sacroiliac joint. The therapist’s forearm was placed on the iliac crest for manipulation along the joint. A high-velocity low-amplitude movement in a caudal to cephalic and posterior to anterior direction was performed to mobilize the sacroiliac and lumbosacral joints. This technique was applied bilaterally [26].

Abdominal mobilization technique: The patient was in the supine position with knees flexed. The ulnar border of both hands of the therapist were placed over the lower part of the abdomen. The patient took a deep breath and, during the exhalation phase, the therapist performed a manual vibration in the cranial direction [27]. This technique was applied for five minutes.

Broad ligament mobilization technique: The patient was in a supine position with knees flexed. The therapist’s cranial hand was placed on the broad ligament while the other hand held the patient’s legs. An opposite movement of both hands was then performed to provoke a stretch in the abdominal region [21]. This technique was applied for five minutes.

Pelvic diaphragm release technique: The patient was in a supine position. One hand was placed under the lumbosacral junction and the other hand on the upper surface of the pelvic area, perpendicular to the one below. A slight compression was performed for 5 min to produce a relaxation in the fascial tissue [28].

Sphenoid technique: The patient was in the supine position. The therapist placed their fingers as follows: the first finger on the midline of the head, the second over the sphenoid (greater wing), the third on the pterion, the fourth behind the ear over the asterion, and the fifth over the occiput (lateral angle). The other hand was placed in the same position on the contralateral side of the head. A gentle caudal to cephalic distraction force was performed for 5 min [29].

Fourth ventricle technique: The patient was in a supine position. The therapist’s hands were placed under the patient’s occiput with the thumb tips together. A slight compression with the thenar eminences and cephalic traction was performed for 5 min [30].

#### 2.4.2. Placebo Treatment

The participants received light contact on the same points and for the same amount of time as the experimental group with no intention to treat [19].

Neither the MTG not the PG were informed of the type of treatment they were receiving. To monitor the blinding and assess if the patients believed that the light contact that they received was normal physiotherapy, both groups were asked to guess which treatment they received at the end of the study [31].

### 2.5. Main Outcome Measures

The demographic responses, anthropometric data, and gynecological characteristics of the participants (time of evolution of endometriosis, pregnancy history, hysterectomy history, endometriosis-associated symptoms, and medication intake) were collected. In order to encourage participants to provide honest responses and avoid social desirability bias, anonymity and confidentiality were ensured throughout the study.

Two physiotherapists were responsible for taking baseline and post-intervention main outcome measurements. In addition, with the goal of standardizing the procedure for data collection, the researchers were trained on the assessment protocol and the use of the measurement tools.

#### 2.5.1. Clinical Characteristics

Pelvic pain intensity was assessed through the Visual Analogue Scale (VAS). It is a commonly used and validated instrument for the interpretation of pain intensity [32]. The scale consists of a 10 cm line divided with numbers from 0 to 10, on which the patient marks the intensity of their pelvic pain (0 = no pain and 10 = worst pain imaginable). It is a valid and reliable pain intensity measurement instrument with an Intraclass Correlation Coefficient (ICC) of 0.97 [32]. The minimum detectable change (MCD) is 1.10 to 1.20 cm. [33].

Lumbar mobility was evaluated using the Schober Test, with modification. In this test, the participant stands in a neutral upright position without shoes and with their feet hip-width apart. A mark is made 5 cm below and 10 cm above the lumbosacral junction (15 cm distance in total). The participant then bends forward to the maximum that they can without bending their knees, and the distance between the marks is measured, with the increase being a measure of anterior flexion. The ICC of the test is 0.77. The MCD is established at 1.8 cm [34].

#### 2.5.2. Clinical Features Related to Quality of Life

The endometriosis health profile was determined using the Endometriosis Health Profile Questionnaire (EHP-30). This questionnaire was comprised of two parts, the first being the core questionnaire, which consisted of five scales (pain, control and powerlessness, emotional wellbeing, social support, and self-image) and contained a total of 30 items. The items within the scales were summed to create a raw score and then each scale was translated into a score ranging from 0 (best health status) to 100 (worst health status) [35]. Cronbach’s α renders a high level of internal consistency reliability for all items of the questionnaire (range: 0.85–0.97) [36]. The minimal important difference is established as 2.0 points [35].

Self-perceived quality of life was assessed using the SF-36 health questionnaire [37]. It is composed of two scales: “physical component”, with four subscales (physical functioning, physical role functioning, bodily pain, and general health perceptions), and “mental component”, with four subscales (vitality, social role functioning, emotional role functioning, and mental health). The total score ranges from 0 to 100, and the higher the score, the better the quality of life. This questionnaire has good reliability (Cronbach’ α = 0.70–0.90) [38]. Effects greater than 12% represent the MCD [39].

#### 2.5.3. Emotional Condition

Depression levels were determined using the Beck Depression Index (BDI-II) [40]. This test consisted of 21 items with four responses (from absent = 0 to severe = 3). The total score ranged from 0 to 63 points. The higher the score, the greater the degree of depression [41]. Internal consistency is described as Cronbach’s α = 0.90, and the test–retest reliability ranges from 0.73 to 0.96 [42]. The MCD for chronic pain has been set at 5 points [43].

Anxiety levels were evaluated through the State Trait Anxiety Index (STAI) [44]. This consists of 40 items divided into two subscales: state-anxiety (items 1 to 20) and trait-anxiety (items 21 to 40). A score of <30 indicates a low anxiety level, while between 30 and 44 points is a medium anxiety level, and >44 points is a high anxiety level. This instrument has a good internal consistency (Cronbach’s α = 0.93) [44], and the reliability for patients with an anxiety disorder ranges from 0.87 to 0.93 [45]. The MCD for chronic pain is 10 points [46].

#### 2.5.4. Patient’s Perception of Change after the Intervention

The patient’s perception of change was assessed using the Patient Global Perception of Change Scale (PGICS). In this scale, the patient reflects on their perception of the efficacy of the intervention on their limitations regarding activities, symptoms, emotions, and overall quality of life. It consists of a 7-point verbal scale, with the options “very much improved”, “much improved”, “minimally improved”, “no change”, “minimally worse”, “much worse”, and “very much worse” [43]. It has excellent test–retest reliability (CCI = 0.90) and is intuitively easy to understand for both the patient and the person administering it [47]. The “Much improvement” item has been reported as clinically relevant in people with chronic pain [48].

### 2.6. Statistics

The statistical analyses were performed using SPSS v.24 (IBM SPSS, Inc., Chicago, IL, USA). The inferential analyses of the data were conducted using a two-factor mixed multivariate analysis of variance (MANOVA), having a between-subjects factor “treatment group” with two categories (MTG and PG), and a within-subject factor “time measurements” with four categories (T0, T1, T2 and T3) for all variables. Bonferroni correction was used for the post-hoc analyses. The assumption of homoscedasticity was determined using Levene’s test, and the sphericity using Mauchly’s test was also evaluated. A Chi-squared test was used for the categorical variables. Additionally, similarity between the groups at baseline was explored using the Student’s *t*-test for the continuous variables and a Chi-squared test for the categorical ones. Cohen’s *d* was computed to analyze the effect size of the continuous variables, which was rated as either small (0.20–0.50), medium (0.50–0.80), or large (>0.80) [49]. The effect size was reported using the contingency coefficient (CC) for the categorical variables.

### 2.7. Sample Size Calculation

For computing the sample size, the software G*Power 3.1.9.7 (Düsseldorf, Germany) was used. We set a power of 80%, an effect size of f^2^ = 0.25, and α error = 0.05. Since this was a study with two intervention groups and four measurement times, a minimum sample size of 12 participants per group (24 participants in total) was generated. Considering possible dropouts, this sample was increased to 20 per group, 40 in total.

## 3. Results

### 3.1. Participants

**Figure 2:**
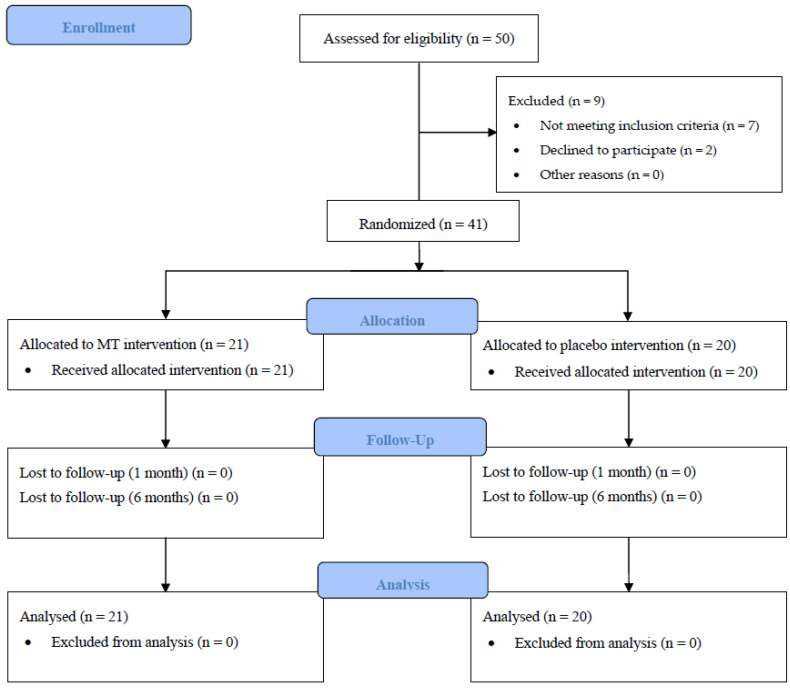
Flowchart according to the CONSORT Statement for the reporting of randomized trials. MT: manual therapy.

Fifty participants were assessed for eligibility, of whom 41 met the inclusion criteria. Following on from this, 21 participants were randomly allocated to the MTG and 20 to the PG. All of them completed the study (Figure 2). There were no serious intervention-related side effects. Table 1 shows the baseline demographic and gynecological characteristics. No significant differences between the groups before the intervention in any variable were shown.

### 3.2. Effect of the Treatment on Pelvic Pain and Lumbar Mobility

As noted in Table 2, there was a significant pain reduction in the MTG at T1 (*p* < 0.001; *d* = 1.00), T2 (*p* < 0.001; *d* = 0.89), and T3 (*p* < 0.001; *d* = 2.28). However, there were no significant differences after the placebo intervention or at the follow-up (*p* > 0.05). In addition, the MTG improved significantly compared to the PG at T1 (*p* = 0.03; *d* = 0.71) and T3 (*p* < 0.001; *d* = 1.81).

Regarding lumbar mobility, although there were no significant differences between the time measurements in the MTG (*p* > 0.05) or in the PG (*p* > 0.05), the MTG significantly showed greater lumbar mobility than the PG at T1 (*p* = 0.001; *d* = 1.17), T2 (*p* = 0.001; *d* = 1.15), and T3 (*p* = 0.004; *d* = 0.99) (Table 2).

### 3.3. Effect of the Treatment on Clinical Features Related to Quality of Life

Regarding the endometriosis health profile assessed with the EHP-30 (Table 3), the intra-group comparisons showed that the MTG significantly improved at T1 for the following items: pain (<0.001; *d* = 1.55), control and powerlessness (*p* = 0.001; *d* = 0.68), emotional wellbeing (*p* = 0.01; *d* = 0.51), and EHP-30 total score (*p* < 0.001; *d* = 0.63). In addition, the changes were maintained in terms of pain at T2 (*p* < 0.001; *d* = 1.12) and T3 (*p* = 0.001; *d* = 1.05), and in the EHP-30 total score at T2 (*p* = 0.01; *d* = 0.46) and T3 (*p* = 0.002, *d* = 0.59). However, there were no significant differences between the time measurements for social support and self-image at T1, T2, or T3 (*p* > 0.05). The PG did not significantly improve any of the EHP-30 items after the intervention and at the follow-up (*p* > 0.05), except for emotional wellbeing at T1 (*p* = 0.04; *d* = 0.65) and T3 (*p* = 0.001; *d* = 0.61). In addition, there were significant differences between the groups in terms of pain (*p* = 0.003; *d* = 0.98) at T1, whereas there were no significant differences between the groups for the items of control and powerlessness, emotional wellbeing, social support, self-image, and the EHP-30 total score at T1, T2, and T3 (*p* > 0.05).

When the quality of life domain was analyzed (Table 3) in the MTG, there were significant changes at T1 in the physical role (*p* = 0.01, *d* = 0.72), corporal pain (*p* = 0.02; *d* = 0.85), general health (*p* = 0.01; *d* = 0.56), and physical subscale (*p* = 0.01; *d* = 0.69) compared to T0. However, there were no changes in the items of physical functioning, vitality, social functioning, emotional role, mental health, mental subscale, and overall quality of life (*p* > 0.05). In addition, these changes were not maintained at either T2 (*p* > 0.05) or T3 (*p* > 0.05). In the PG, only the mental health item was significantly increased at T2 (*p* = 0.03; *d* = 0.42). However, no significant changes were found at T1, T2, or T3 for the remaining items (*p* > 0.05). Regarding the between-groups comparisons, the MTG significantly improved the corporal pain item compared to the PG at T1 (*p* = 0.03; *d* = 0.71), although not at T2 (*p* > 0.05) or T3 (*p* > 0.05). For the other items, there were no significant differences between the groups at T1, T2, or T3 (*p* > 0.05).

### 3.4. Effect of the Treatment on Emotional Condition

When the participant’s mental status was analyzed, there were no significant changes between the time measurements T1, T2, or T3 compared to T0 in either of the two groups (i.e., MTG and PG), nor between the groups at T1, T2, or T3 in terms of depression, state-anxiety, trait-anxiety, and overall anxiety (*p* > 0.05) (Table 4).

### 3.5. Patient’s Perception of Change after Treatment

**Figure 3:**
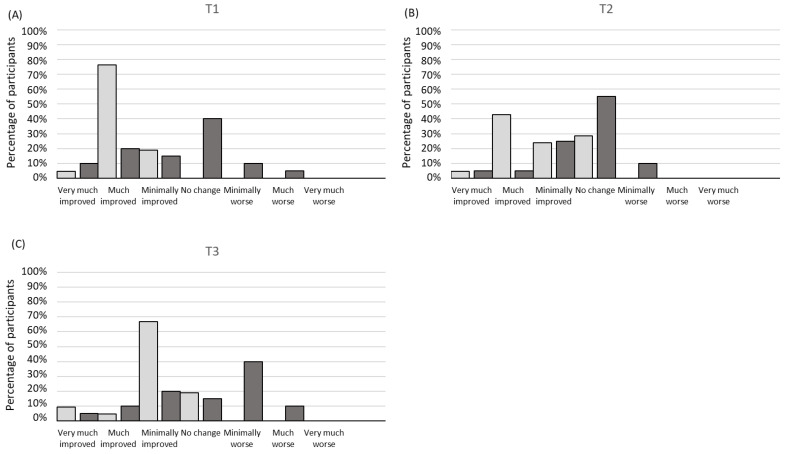
Patient’s perception of change after treatment at T1 (**A**), T2 (**B**), and T3 (**C**). The Manual Therapy group (MTG) is represented by light gray bars, whereas the Placebo group (PG) is represented by dark gray bars.

The MTG reported a greater perception of change than the PG at T1 (χ^2^ (5) = 18.66; *p* = 0.002; CC = 0.56) and T3 (χ^2^ (5) = 14.80; *p* = 0.01; CC = 0.52) (Figure 3). However, there were no significant differences between the groups in terms of the perception of change at T2 (*p* > 0.05).

### 3.6. Patients’ Perception of Group Assignment

All the participants (100%) reported that the treatment they received was normal physiotherapy at the end of the study, regardless of the assigned group (Table 5).

## 4. Discussion

The present study shows that a protocol based on manual therapy techniques improves pelvic pain, the endometriosis health profile, and physical quality of life. Interestingly, changes in pelvic pain were maintained after both the one- and six-month follow-ups. Furthermore, significant differences were observed in terms of pain and lumbar mobility at T1, T2, and T3 compared to the placebo intervention. However, the proposed protocol had no effect on depression and anxiety levels.

Pelvic pain is one of the most common symptoms in women with endometriosis, and it often persists after medical treatment [7]. Our results show that a manual therapy protocol combining soft tissue and articulatory techniques reduced pain by 30.76% after a six-week intervention and by 27.26% after a one-month follow-up in people with endometriosis-related pelvic pain. Several factors have been proposed to be involved in the analgesic effects of manual therapy. In this regard, it has been suggested that soft tissue techniques improve the quality of the muscle–fascial structures and connective tissue, and that it also favors blood circulation and drainage in women with gynecological problems [50,51]. Furthermore, manual therapy has been reported to trigger a neurophysiological response associated with pain descending pathway modulation, together with an endogenous opioid response and reduction in inflammatory markers [52,53], decreased spinal excitability and pain sensitivity [54], the excitation of the sympathetic nervous system, and changes in motor function [55,56]. A local and widespread analgesic effect, as assessed using an algometer, has been described following spinal or peripheral manual techniques [57]. Furthermore, a previous study applied an osteopathic treatment consisting of the release of the pelvic and abdominal diaphragms, abdominal mobilization techniques, and cranial techniques in women with chronic pelvic pain due to endometriosis [23]. After the 4-week intervention, the participants perceived a decrease in pain of 53.01%. In contrast to the present study, they did not analyze long-terms effects, which is recommended when studying the effects on endometriosis symptomatology, to better understand its efficacy and safety over time, and to make informed decisions about treatment [58].

Lumbar mobility and pain are closely related, as pain can limit a person’s ability to move. Furthermore, chronic fatigue and pain-catastrophizing thoughts have been related to physical limitations in the activities of daily living of women with endometriosis [59]. Additionally, this population usually presents with physical deconditioning and a lack of musculoskeletal flexibility [12]. The results of the present study show that the MTG significantly increased lumbar spine movement after the intervention and at the follow-up assessments compared to the PG. This can be explained by the improvement in pain and physical health in the MTG, since the person may feel more comfortable moving and performing the activities that they previously avoided due to pain [59]. In addition, pain is often associated with inflammation and muscle tension in the affected area. By treating the underlying cause of the pain, inflammation and muscle tension may have been reduced. This may have led to a greater flexibility and range of motion in the lumbar area, which, in turn, may have improved lumbar mobility [60]. Unfortunately, the results with regard to lumbar mobility observed in this study cannot be compared with previous studies, since this is the first study to determine the effect of manual therapy on this variable in people with endometriosis.

Regarding the endometriosis health profile measured with the EPH-30, an improvement in control and powerlessness and in emotional wellbeing was observed. In line with these findings, a recent review concluded that pelvic physiotherapy stimulates the self-empowerment of women and supports the recovery of function that they may have lost due to pain and dysfunction [13]. However, although the MTG effect size was larger, emotional wellbeing also improved after the placebo intervention. Since the placebo intervention was not intended to treat, this improvement may be explained by the “hands on” effect and the feeling of being treated [61]. However, there were no significant changes in social support and self-image in either of the two groups. Previously, Goyal et al. [23] carried out a study involving 29-year-old women with endometriosis who received eight sessions of an osteopathic treatment. After the intervention, they observed an improvement in their endometriosis health profile, measured with an EPH-5 of 72 to 26 points out of 100. Nevertheless, the findings cannot be generalized, since it was a case report.

Pain and associated gynecological symptoms, such as burning during urination (dysuria) and pain during sexual intercourse (i.e., dyspareunia), have a great impact on the patients’ quality of life [4]. After the intervention, the MTG experienced an improvement of 28.54% in physical quality of life, with significant changes in physical role, corporal pain, and general health. In agreement with the current study, an improvement in physical quality of life was found after the application of the abdominal mobilization technique, broad ligament mobilization technique, and thoraco-lumbar manipulation technique in women with deep infiltrating endometriosis and colorectal involvement [21,62]. However, they reported greater changes in their mental quality of life compared to ours after the intervention. It should be noted that the duration (in minutes and number of sessions) of their protocol was not standardized, which makes it difficult to replicate the intervention. In addition, they did not compare the results with a control or placebo comparator group. This is of importance, since a psychological change in the patient, caused by the attribution of significance to the treatment, can be developed [63].

Symptoms of depression and anxiety occur frequently in endometriosis patients and are related to chronic pain [64]. However, to our knowledge, this is the first study to assess the effects of any type of manual therapy. The results show that there were no significant improvements in depression and anxiety levels after the intervention or at the follow-up measurement times. This is in accordance with a study that applied a manual therapy protocol for chronic pain [20]. The lack of significant changes can be explained because, together with physical therapy, it would be convenient to combine it with cognitive behavioral therapy, pain biology education, mindfulness-based stress reduction, yoga, and imagery exercises, to address the biopsychosocial components of female sexual pain [65].

Another important variable that has gained attention in the field of chronic pain is the patient’s perception of change [43]. In this regard, 76.19% of the MTG participants reported that they improved much at T1, while 42.86% found that these improvements were maintained at T2. These improvements may be explained by the improvement found for pelvic pain [48]. These results are in agreement with a previous study in which 71.43% of women with endometriosis reported symptom improvement after a 6-week intervention based on osteopathic treatment with spinal manipulations in the cervical region, thoraco-lumbar region, and sacroiliac joints, as well as soft tissue techniques such as the abdominal mobilization technique [22]. However, since it was a pilot non-controlled study limited by a small population size, the results could not be generalized.

This study had some limitations. First, since the manual therapy protocol included a variety of techniques, the improvement cannot be entirely attributed to just one of them. Second, since patients were permitted to use analgesic and anti-inflammatory medication as needed throughout the process, future studies should monitor whether this influenced the results with regard to the pain variable over time. Third, only pre-menopausal woman were included, yet women of reproductive age are those that suffer from the greatest impact [1]. Additionally, we focused on women with endometriosis (regardless of the clinical form and severity of endometriosis or disease evolution) and associated pelvic pain, which jeopardizes the generalizability of the results. Indeed, the severity of endometriosis and disease evolution (although not being statistically significantly different) slightly differed between groups. Thus, further studies should ascertain whether different clinical forms and characteristics of endometriosis result in different responses to treatment. In addition, the participants were recruited from the primary care centers of one city; thus, more studies applying this protocol in other populations and in relation to other types of chronic pelvic pain-related pathologies are needed to generalize the results. Furthermore, another limitation to the generalizability of the results is the extensive clinical practice of the physiotherapist. Moreover, in the current study, no objective outcome measures were employed, as only questionnaires, scales, and the Schober test were applied. Therefore, future studies should use objective assessment tools to verify the obtained results. Moreover, the effects of personality and personal patient–physiotherapist interactions were challenging to monitor. However, all the participants were treated by the same physiotherapist to avoid differences in their interactions. Furthermore, the emotional properties that emerge from the sympathetic contact established with the patient cannot be controlled because they are specific effects of manual therapy that occur in daily clinical practice [66]. Finally, the Hawthorne effect during the study was not controlled, as the mere presence of an observer has the potential to influence human behavior.

## 5. Conclusions

Without waiving the limitations mentioned above, a manual therapy protocol based on soft tissue and articulatory techniques is effective at reducing pain and improving women’s quality of life but not the emotional status of women with endometriosis; hence, it may be considered a valid tool for improving pelvic pain due to endometriosis.

## Figures and Tables

**Table 1:** Baseline demographic and gynecological characteristics.

	MTG (*n* = 21)	PG (*n* = 20)	Total (*n* = 41)	*p*-Value
Age ^a^	34.85 (7.23)	37.40 (6.62)	36.10 (6.97)	0.25
Time of evolution (months) ^a^	38.86 (43.53)	74.18 (88.02)	56.09 (70.34)	0.11
Pregnancy history ^b^				
No pregnancy	10 (47.62%)	10 (50.00%)	20 (48.78%)	0.88
One pregnancy	7 (33.33%)	6 (30.00%)	13 (31.71%)	0.82
More than one pregnancy	2 (9.52%)	2 (10.00%)	4 (9.76%)	0.96
Miscarriage	2 (9.52%)	3 (15.00%)	5 (12.20%)	0.59
Delayed fertility	6 (28.57%)	4 (20.00%)	10 (24.39%)	0.52
Hysterectomy history ^b^				0.07
No	21 (100.00%)	17 (85.00%)	38 (92.68%)	
Yes	0 (0.00%)	3 (15.00%)	3 (7.32%)	
Symptoms associated with endometriosis ^b^				
Abdominal swelling	17 (80.95%)	16 (80.00%)	33 (33.00%)	0.94
Menstrual pain	15 (71.43%)	11 (55.00%)	26 (26.00%)	0.28
Non-menstrual pelvic pain	15 (71.43%)	15 (75.00%)	30 (30.00%)	0.80
Fatigue	14 (66.67%)	11 (55.00%)	25 (25.00%)	0.44
Low back pain	13 (61.90%)	13 (65.00%)	26 (26.00%)	0.84
Ovulation pain	11 (52.38%)	5 (25.00%)	16 (16.00%)	0.07
Dyspareunia	8 (38.10%)	10 (50.00%)	18 (18.00%)	0.44
Abundant bleeding	8 (38.10%)	7 (35.00%)	15 (15.00%)	0.84
Diarrhea	8 (38.10%)	7 (35.00%)	15 (15.00%)	0.84
Constipation	8 (38.10%)	13 (65.00%)	21 (21.00%)	0.09
Urinary incontinence	4 (19.05%)	6 (30.00%)	10 (10.00%)	0.414
Full bladder sensation	4 (19.05%)	8 (40.00%)	12 (12.00%)	0.141
Delayed fertility	2 (9.52%)	3 (15.00%)	5 (5.00%)	0.592
Infertility	1 (4.76%)	2 (10.00%)	3 (3.00%)	0.520
Medication intake ^b^				
Hormonal	9 (42.86%)	13 (65.00%)	22 (53.66%)	0.16
Analgesics	15 (71.43%)	13 (65.00%)	28 (68.29%)	0.66
Anti-inflammatory	3 (14.29%)	2 (10.00%)	5 (12.20%)	0.68
Dietary supplements	3 (14.29%)	3 (15.00%)	6 (14.63%)	0.95
Anxiolytic/Antidepressant	2 (9.52%)	0 (0.00%)	2 (4.88%)	0.16
None	4 (19.05%)	2 (10.00%)	6 (14.63%)	0.41

Data shown as ^a^ mean (standard deviation) and ^b^ frequency (percentage). MTG: Manual Therapy group; PG: Placebo group.

**Table 2:** Effect of the treatment on pelvic pain and lumbar mobility.

Variable	Group	T0	T1	T2	T3
Pain (VAS)	MTG	5.43 (1.78)	3.76 (1.55) * #	3.95 (1.86) *	1.62 (1.56) * #
	PG	5.10 (1.92)	5.05 (2.09)	4.70 (2.08)	4.55 (1.67) *
Lumbar mobility (Schober test; cm)	MTG	19.62 (1.06)	20.05 (0.99) #	20.08 (0.94) #	19.94 (1.38) #
	PG	19.00 (1.48)	19.04 (0.73)	19.13 (0.71)	18.08 (2.39)

Data shown as mean (standard deviation). MTG: Manual Therapy group; PG: Placebo group; T0: baseline; T1: post-intervention; T2: one-month follow-up; T3: six-month follow-up. VAS: Visual Analogue Scale. *: *p* < 0.05 compared to baseline; #: *p* < 0.05 compared to PG.

**Table 3:** Effect of the treatment on the clinical features related to quality of life.

Variable	Group	T0	T1	T2	T3
Endometriosis Health Profile (EHP-30)					
Pain	MTG	52.38 (16.17)	25.87 (17.96) * #	30.12 (23.65) *	30.95 (24.49) *
	PG	47.27 (16.43)	42.05 (15.10)	38.18 (20.76)	36.14 (23.93)
Control and powerlessness	MTG	48.81 (22.09)	33.44 (22.90) *	41.83 (28.04)	38.29 (25.87)
	PG	48.54 (19.97)	42.84 (14.11)	41.04 (25.26)	42.50 (27.92)
Emotional wellbeing	MTG	55.75 (17.70)	44.44 (26.63) *	48.21 (24.32)	41.47 (22.11) *
	PG	47.50 (13.75)	37.50 (17.15) *	38.75 (14.44)	37.92 (17.46) *
Social support	MTG	53.87 (20.39)	47.92 (33.68)	47.62 (26.55)	47.02 (30.47)
	PG	45.31 (27.72)	39.06 (16.95)	42.38 (22.31)	39.06 (22.39)
Self-image	MTG	47.62 (21.11)	44.44 (30.43)	46.03 (28.58)	42.06 (29.87)
	PG	45.42 (22.70)	40.00 (25.01)	40.00 (24.42)	45.42 (27.24)
Total score	MTG	51.69 (16.01)	39.22 (23.56) *	42.76 (22.50) *	39.96 (23.66) *
	PG	46.81 (14.76)	40.29 (14.22)	40.07 (17.39)	40.21 (20.13)
Quality of life (SF-36)					
Physical functioning	MTG	83.57 (21.28)	84.52 (21.21)	80.95 (22.28)	90.24 (10.78)
	PG	72.75 (26.28)	78.00 (21.30)	76.25 (26.40)	78.50 (29.43)
Physical role	MTG	30.95 (42.50)	60.71 (40.75) *	50.00 (41.08)	52.38 (41.01)
	PG	42.50 (45.23)	48.75 (40.13)	50.00 (45.16)	50.00 (45.88)
Corporal pain	MTG	48.57 (24.68)	64.00 (23.82) * #	62.67 (20.54)	55.81 (25.46)
	PG	42.30 (22.74)	46.40 (25.60)	49.00 (26.43)	51.30 (30.53)
General health	MTG	39.81 (19.85)	51.62 (22.39) *	44.52 (18.72)	46.05 (20.48)
	PG	39.50 (15.70)	43.25 (17.54)	47.50 (20.84)	44.25 (19.67)
Vitality	MTG	40.00 (20.86)	48.57 (20.13)	47.14 (18.55)	46.19 (19.93)
	PG	46.25 (20.89)	46.75 (21.78)	48.00 (19.76)	48.75 (19.79)
Social functioning	MTG	67.26 (22.87)	67.26 (29.97)	74.40 (37.18)	61.90 (28.63)
	PG	62.50 (25.33)	69.38 (26.74)	70.00 (26.72)	71.88 (30.31)
Emotional role	MTG	44.44 (45.13)	65.08 (37.23)	60.32 (37.45)	63.49 (42.04)
	PG	68.33 (43.90)	71.67 (34.67)	66.67 (40.47)	66.67 (34.20)
Mental health	MTG	55.62 (15.28)	61.52 (14.06)	56.57 (11.19)	59.62 (17.34)
	PG	58.20 (17.96)	67.20 (17.15)	66.00 (18.87) *	66.60 (18.32)
Physical subscale	MTG	50.73 (20.63)	65.21 (21.29) *	59.54 (19.30)	61.12 (20.25)
	PG	49.26 (22.91)	54.10 (20.97)	55.69 (26.33)	56.01 (27.7)
Mental subscale	MTG	51.83 (22.01)	60.61 (22.14)	58.27 (17.62)	57.80 (23.51)
	PG	58.82 (20.48)	63.75 (20.58)	62.67 (21.24)	63.47 (18.65)
Overall quality of life	MTG	51.28 (20.12)	61.00 (22.53)	60.03 (19.66)	59.46 (20.53)
	PG	54.04 (20.23)	59.71 (19.68)	59.33 (22.98)	59.74 (21.59)

Data shown as mean (standard deviation). MTG: Manual Therapy group; PG: Placebo group; T0: baseline; T1: post-intervention; T2: one-month follow-up; T3: six-month follow-up. EHP-30: Endometriosis Health Profile. SF-36: Short Form Health Questionnaire. *: *p* < 0.05 compared to baseline; #: *p* < 0.05 compared to PG.

**Table 4:** Effect of the treatment on depression and anxiety levels.

Variable	Group	T0	T1	T2	T3
Depression levels (BDI-II)	MTG	15.86 (8.45)	13.71 (9.32)	13.29 (8.85)	11.14 (10.07)
	PG	14.90 (10.20)	13.20 (8.73)	12.10 (7.58)	9.60 (6.98)
Anxiety levels (STAI)					
State-anxiety	MTG	36.14 (5.52)	45.00 (13.94)	43.57 (12.50)	46.71 (14.84)
	PG	37.70 (3.37)	41.70 (10.15)	39.90 (8.03)	39.40 (7.79)
Trait-anxiety	MTG	40.57 (10.03)	38.57 (12.42)	37.57 (13.46)	41.29 (11.76)
	PG	41.30 (7.44)	35.40 (9.17)	36.80 (7.64)	37.70 (8.38)
Overall anxiety levels	MTG	38.36 (6.54)	41.79 (12.93)	42.79 (10.82)	44.00 (13.22)
	PG	39.50 (4.43)	38.55 (9.26)	38.40 (7.55)	38.55 (7.92)

Data shown as mean (standard deviation). MTG: Manual Therapy group; PG: Placebo group; T0: baseline; T1: post-intervention; T2: one-month follow-up; T3: six-month follow-up BDI-II: Beck Depression Inventory; STAI: State Trait Anxiety Index.

**Table 5:** Patients’ perception of group assignment at T2.

	MTG (*n* = 21)	PG (*n* = 20)
Normal physiotherapy	21 (100%)	20 (100%)
Control group	0 (0%)	0 (0%)
Others	0 (0%)	0 (0%)

Data shown as frequency (percentage). MTG: Manual Therapy group; PG: Placebo group.

## Data Availability

The data presented in this study are available on request from the corresponding author. The data are not publicly available due to their containing information that could compromise the privacy of research participants.
